# Invisible conflicts: the impact of covert academic bullying among “Double First-Class” young medical teachers on academic output—a qualitative study

**DOI:** 10.3389/fpsyg.2026.1712457

**Published:** 2026-01-20

**Authors:** Cheng Yao, Zaihao Wu, Siliang Yu

**Affiliations:** 1 Xihua University, Chengdu, China; 2 Nakhon Phanom University, Nakhon Phanom, Thailand; 3 Sichuan Normal University, Chengdu, China

**Keywords:** academic output, chain-exclusion mechanism, covert academic bullying, Double First-Class universities, young medical teachers

## Abstract

**Background:**

China’s “Double First-Class” initiative has significantly reshaped higher education resources. While medical disciplines benefit from concentrated funding and platforms, they also face heightened competition and pressure. This study investigates the manifestations and mechanisms of covert academic bullying in these universities and its full-chain impact on young medical teachers’ academic output.

**Methods:**

Using phenomenological and social constructivist approaches, 21 teachers from 12 universities were recruited through purposive and snowball sampling for semi-structured interviews. Data were transcribed, anonymized, and thematically analyzed with NVivo, yielding six core themes.

**Results:**

Covert academic bullying follows an “entry restriction–process constraint–endpoint undermining” chain, involving information withholding, institutional marginalization, resource deprivation, heavy workload, unfair authorship, and reputation manipulation. Gender and career stage intersections intensify disadvantages, while core teams’ monopolization of resources restricts peripheral teachers’ opportunities in collaboration, project access, and platform utilization.

**Conclusion:**

The phenomenon is persistent and linked to centralized management and relationship-based culture. Recommended interventions include improving information transparency, ensuring review fairness, securing independent budgets and priority resources, and protecting authorship and reputation, supported by cross-institutional cooperation and online platforms to sustain research productivity and career resilience.

## Introduction

Over the past decade, China’s Double First-Class (DFC) initiative, designed to develop world-class universities and disciplines, has significantly transformed the allocation of resources and the ecosystem of research talent within higher education. Since 2016, the government has invested approximately 167 billion RMB in DFC institutions to sustain academic excellence via a framework comprising high-level discipline construction, high-level research output, and global competitiveness (Araya and Peters, 2023). By 2022, the number of DFC universities reached 147, including numerous medical institutions and key disciplines (Zheng and Li, 2024). Medical disciplines, which bear the combined responsibilities of research, teaching, and clinical service, have benefited from the concentration of platforms and funding, while also facing increasing metric-driven competition and peer pressure.

Within DFC medical universities, the early stages of young medical teachers’ (YMTs) academic careers are especially pivotal. Access to experimental platforms, opportunities for cross-faculty collaboration, authorship rules, and evaluation criteria directly influence their short-term performance and long-term academic identity (Chai and Wu, 2025; Bowrey et al., 2024). However, these opportunities are not fully guaranteed by formal systems. Certain pivotal processes are shaped by implicit rules that avoid external scrutiny, giving rise to a hidden, system-embedded conflict known as covert academic bullying (CAB). Distinct from overt public criticism or direct personal attacks, CAB is frequently embedded in routine research management and collaborative practices (Noakes and Noakes, 2021). Manifestations include exclusion from project and funding allocation, imposition of excessive, non-research-related duties, deliberate delay or withholding of critical information, manipulation of authorship rights, ambiguity in evaluation criteria, and everyday social isolation (Averbuch et al., 2021; Einarsen et al., 2020).

International research shows that excessive workload is the most prevalent form of bullying in academic medicine (38.2%), with senior physicians or consultants accounting for the majority of perpetrators (53.6%). Only 28.9% of victims opt to file formal reports, and more than half of these cases remain unresolved (57.5%) (Averbuch et al., 2021). Broader surveys reveal a strong connection between competitive culture, mental health, and academic productivity: 78% of researchers believe that excessive competition creates an unfriendly and aggressive environment, 61% have witnessed bullying or harassment, 43% have personally experienced it, and 53% have sought or contemplated mental health support (Wellcome, 2020). These findings suggest that when quantifiable research metrics dominate evaluation systems, informal power is more prone to influence collaborative processes, thereby undermining young scholars’ research opportunities, psychological safety, and sustained productivity, while also posing long-term risks to research quality, ethics, reproducibility, and clinical translation efficiency.

China is implementing research evaluation reforms to address assessment biases. However, inconsistent faculty-level regulations and uneven enforcement can still be exploited by certain power holders, giving rise to or exacerbating CAB in areas such as resource allocation, performance evaluation, and hierarchical relationships (Zhu and Du, 2024; Shu et al., 2022). Within the medical field’s distinctive “apprenticeship–hierarchical chain–platform dependence” structure (Zhou et al., 2024; Liu et al., 2023), YMTs typically face high entry barriers to accessing equipment, samples, data, and interdisciplinary collaborations, and are more dependent on discipline leaders or core collaborators (Averbuch et al., 2021). This structure is compounded by the high concentration, intense competition, and stringent constraints characteristic of DFC medical disciplines. When the scarcity of research outcomes combines with ambiguous and asymmetric evaluations, it tends to lead to conservative research agendas, avoidance of innovation, erosion of team trust, and an accumulation of risks to reproducibility and compliance.

From a theoretical standpoint, foundational theories in organizational psychology and labor sociology offer essential frameworks for analyzing implicit conflicts in academic settings. For example, existing research conceptualizes workplace bullying as a systematic and recurrent process embedded in organizational structures, rather than as isolated interpersonal incidents, highlighting the influence of hierarchy, informal power, and institutional dynamics (Pauksztat and Salin, 2020). Moreover, resource dependence theory argues that control over critical research resources generates power asymmetries and limits individual agency, a mechanism especially pronounced in highly centralized and competition-oriented academic environments (Hillman et al., 2009).

In recent years, international attention to CAB has steadily increased. Several universities, including the University of Cambridge in the United Kingdom, have reported dissatisfaction with the handling of bullying and harassment cases, prompting debates over governance transparency and accountability mechanisms (The Guardian, 2025). In China, awareness of covert conflicts has gradually increased, providing a foundation for institutional interventions and cultural reshaping. This study uses the daily experiences of YMTs in DFC medical universities as the focal point, systematically examining their encounters with CAB and its impact on academic productivity, to provide empirical evidence for the formulation of governance strategies that are both institutionally feasible and culturally sensitive.

## Methods

### Research design

This qualitative study aims to examine the mechanisms of CAB experienced by YMTs in DFC medical universities and to evaluate its long-term impact on academic productivity. Grounded in a phenomenological approach, the study explores the implicit connections between power imbalance, resource allocation, and academic competition through teachers’ lived experiences, and applies a social constructivist framework to analyze how these dynamics are perpetuated within the academic community. CAB is a relational form of organizational conflict, often manifested through resource deprivation, social exclusion, information blocking, and institutional marginalization, characterized by concealment and contextual dependence (Scheeler et al., 2022), and is difficult to capture through quantitative research. To investigate this phenomenon, open-ended semi-structured interviews were conducted in 12 DFC medical universities, using purposive and snowball sampling to recruit participants with diverse backgrounds. The findings offer empirical evidence for subsequent mechanism analysis and the development of intervention strategies.

### Participants

The study included current faculty members from 12 DFC medical universities in China. According to Creswell and Poth (2018), thematic saturation in qualitative research is typically achieved with 15–20 participants. A total of 21 participants were ultimately recruited for this study. The inclusion criteria were: (1) under the age of 35; (2) at least 3 years of work experience; (3) documented experience of CAB within the past 2 years (e.g., exclusion, isolation, information withholding, or resource deprivation); (4) voluntary participation accompanied by signed informed consent; and (5) agreement to audio-recorded interviews. Participants holding senior administrative positions were excluded to minimize bias arising from managerial roles. Purposive and snowball sampling were used until thematic saturation was achieved (Baker and Edwards, 2012; Hennink et al., 2017).

The demographic characteristics of the participants are summarized in Table 1. The study included 21 YMTs (mean age = 32.9 years) with an average teaching experience of 7.3 years. Six participants (28.6%) were male and fifteen (71.4%) were female. Most participants held lecturer positions (90.5%), and two held associate professor positions. In terms of disciplinary distribution, eight specialized in basic medicine, six in pharmacy, five in clinical medicine, and two in nursing. More than 76.2% (16/21) of participants had not engaged in academic rank promotion or research project application within the past 2 years.

**Table 1:** Demographic and professional characteristics of the participants (*n* = 21).

Characteristic	Category	*n*	%
Gender	Male	6	28.6
	Female	15	71.4
Age	28–31 years	7	33.3
	32–35 years	14	66.7
Teaching experience	≤5 years	10	47.6
	6–10 years	11	52.4
Academic rank	Lecturer	19	90.5
	Associate Professor	2	9.5
Discipline	Basic Medicine	8	38.1
	Pharmacy	6	28.6
	Clinical Medicine	5	23.8
	Nursing	2	9.5
Promotion/project application in past 2 years	Yes	5	23.8
	No	16	76.2

### Data collection

Data collection was conducted from June to August 2025. The majority of interviews were conducted in independent meeting rooms on university campuses, and participants who could not attend in person due to geographical constraints were interviewed remotely via an encrypted video platform (Tencent Meeting). A semi-structured, in-depth interview approach was employed. Based on a review of relevant literature, a preliminary interview guide was designed and tested in two pilot interviews (one associate professor and one lecturer). Feedback from these pilots informed the refinement of the sequence and phrasing of questions, resulting in the final interview guide.

All interviews were conducted by a trained researcher, with another team member responsible for taking notes. Informed consent was obtained prior to each interview, and all sessions were audio-recorded in Mandarin, each lasting approximately 60–90 min. Participants were coded sequentially from Z1 to Z21 according to the alphabetical order of participants’ names in pinyin. Data collection continued until thematic saturation was reached, defined as the point at which no additional key information emerged. At the end of each day, the research team held debriefing meetings to review the process, exchange preliminary insights, and discuss any issues and potential improvements.

### Data analysis

This study adopted a reflexive, inductive thematic analysis approach in accordance with the six phases proposed by Braun and Clarke (2006): (1) familiarization with the data, (2) generating initial codes, (3) searching for themes, (4) reviewing themes, (5) defining and naming themes, and (6) producing the report. All interviews were transcribed verbatim by members of the research team within 24 h of completion and anonymized. The transcripts were organized, reviewed, and proofread to ensure accuracy.

Two researchers independently conducted the coding process and compared their results, and reached consensus through discussion. When necessary, a third researcher was consulted to resolve disagreements. To ensure systematic and traceable analysis, NVivo software (version 15.1.1) was used for data management and coding, which facilitated the classification, retrieval, and thematic aggregation of large volumes of textual data. The analysis identified six core themes: information withholding, institutional marginalization, resource deprivation, excessive workload, unfair authorship, and control of discourse and reputation.

### Ethics approval

The study received approval from the Ethics Committee of Sichuan Normal University. All procedures adhered to the Declaration of Helsinki (World Medical Association, 2025). Written informed consent was obtained from all participants after they were provided with comprehensive information about the study, and they were informed of their right to withdraw at any time. All recordings, transcripts, and analytical materials were anonymized and used exclusively for research purposes.

## Results

**Figure 1:**
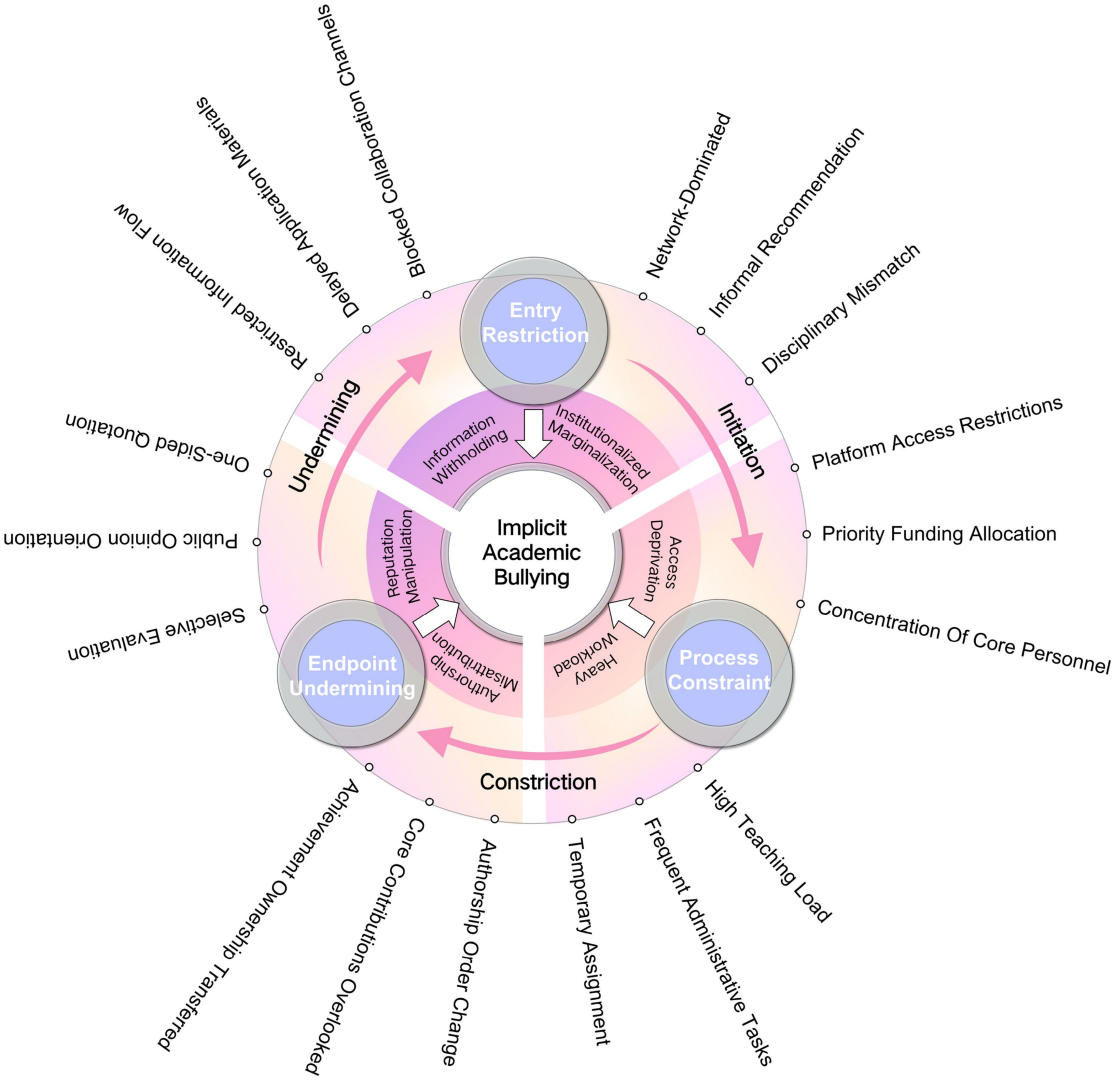
Covert academic bullying chain-exclusion model.

Findings from the interviews and qualitative coding suggest that CAB constitutes a long-term structural phenomenon shaped by the interplay of multiple mechanisms, whose influence permeates all critical stages of research output. Although these behaviors are often framed as academic collaboration or institutional enforcement, they fundamentally alter the conditions of competition, entrench power structures, and generate cumulative adverse effects over time. From the analysis, six interrelated themes were identified (see Figure 1).

### Theme 1: information withholding

In the early stages of research project initiation, access to key information directly determines eligibility to participate in academic competition. Interview findings indicate that in some DFC medical universities, project announcements, collaboration opportunities, and data resources are often circulated within closed “small circles,” relying primarily on informal relationships rather than open channels. Delays or absences in information dissemination result in the exclusion of certain YMTs from competition at the outset of a project. Time lag was identified as the most covert entry barrier.

A YMT who had recently been promoted from teaching assistant to lecturer recalled: *“When I learned about the call for a provincial key project, there were only three days left before the deadline. Although I managed to complete the application materials, ensuring quality and competitiveness was almost impossible.”* (Z8, Lecturer). Such delayed information technically leaves peripheral members eligible to apply but, in practice, undermines their competitiveness.

In some cases, information inequality manifests as an institutionalized “semi-transparency.” One participant with 4 years of research experience noted: *“Some key laboratories give advance internal notices to closely connected teams, while the official announcement is released at the same time for everyone. I once encountered an open call from a national-level platform that seemed public, but by the time the notice was issued, most slots had already been allocated.”* (Z2, Lecturer). This approach maintains formal fairness in policy yet confers advantages in execution.

Several participants further emphasized that opportunities for research collaboration are frequently communicated through specific relationship networks and informal verbal exchanges. This mode of information sharing restricts access to core information, placing peripheral members at a disadvantage in resource acquisition:

*“Some collaboration opportunities are just casually mentioned in meetings or whispered during coffee breaks. People like us, who are not in the circle, only learn about them afterward and never receive any formal notice.”* (Z5, Lecturer).

*“Cross-faculty data collaborations generally require someone to make the introduction. If you are not part of that circle, you won’t even have the chance to sit down and talk, let alone participate.”* (Z16, Lecturer).

Information withholding thus serves as a critical entry point for CAB. This “small-circle” mode of information circulation significantly reduces peripheral YMTs’ chances of entering high-impact projects and interdisciplinary collaborations, thereby diminishing their potential to generate outputs for high-impact journals and large-scale research initiatives.

### Theme 2: institutionalized marginalization

Nearly all participants reported experiencing institutionalized marginalization, particularly evident in processes including academic rank evaluation, research project selection, and resource allocation, where hidden barriers operate. Although relevant rules appear open and transparent in policy documents, their practical implementation is often shaped by relationship networks and informal recommendation mechanisms, placing some YMTs at a sustained disadvantage in long-term competition. One participant who had repeatedly failed in selection processes stated:

*“During evaluations, certain teams always seem to know the reviewers’ preferences in advance. We cannot even get a slot to submit our materials. Even though the rules are clearly written, when it comes to actual implementation, the shortlist and final results tend to favor the same familiar names.”* (Z11, Lecturer).

Some YMTs meet both research and teaching benchmarks yet are marginalized because they are not affiliated with core teams. As one experienced teaching faculty member reported: *“My research and teaching indicators have already met the requirements, and I have continuously published in core journals, but because I am not part of a few core project groups, I was advised during the evaluation to ‘gain a few more years of experience.’”* (Z21, Lecturer).

The causes of institutionalized marginalization are not necessarily attributable to insufficient research capability. In some cases, they are closely tied to the degree to which a discipline aligns with macro-level policy priorities. For example, one participant noted that their research field had seldom appeared in the national priority support list over the past few years, resulting in a lack of reviewers familiar with the area: *“The value of the project is systematically underestimated in the absence of effective evaluation. Even if the quality of the application materials is high, it is difficult to secure support in fierce competition.”* (Z10, Associate Professor).

Such circumstances delay promotion, leading YMTs to shift their focus toward routine teaching and administrative tasks, thereby reducing their opportunities to engage in high-level projects. Over time, highly innovative and interdisciplinary research encounters greater barriers to project approval and career advancement, fostering a tendency toward low-risk, low-impact research topics, which weakens research quality and academic diversity and ultimately reduces international competitiveness.

### Theme 3: access deprivation

Beyond institutional barriers, access deprivation is among the most direct and frequent manifestations of CAB. Participants consistently reported that critical research resources, including funding, clinical trial conditions, affiliated hospital resources, and high-quality graduate students or research assistants, are frequently influenced by relationship networks, informal coordination, and the preferences of decision-making bodies during allocation and use. This results in implicit resource monopolization and an unequal competitive environment. Most participants reported encountering such situations and regarded them as persistent sources of stress in advancing their research.

One participant recounted a case of diverted funding: *“I had successfully secured a university-level key project, but less than two months after the funds were credited, they were reallocated to another team on the grounds that they needed it more urgently. My research had to be suspended as a result.”* (Z1, Lecturer). Another described exclusive control over an experimental platform: *“My team booked a large-scale experimental platform a month in advance, but just before we were scheduled to use it, I was informed that our slot was canceled and reassigned to a research group with close ties to the school leadership.”* (Z14, Lecturer).

Access deprivation also includes competition for research personnel. One participant reported considerable frustration: *“Last year, a highly skilled clinical student was admitted as my master’s student, with the plan to have them take charge of core data collection. However, a week before enrollment, the student was poached by another supervisor, making it impossible to implement the original data collection plan and forcing an extension of the research timeline.”* (Z12, Associate Professor).

For researchers dependent on affiliated hospital case resources, access deprivation frequently produces cascading effects. As one lecturer explained, even if the current project could be completed using alternative data, the sequence of data accumulation for subsequent studies would be disrupted by the loss of key cases (Z3, Lecturer).

### Theme 4: heavy workload

In DFC universities, junior faculty members are often required to undertake disproportionately heavy workloads. Prolonged high-intensity demands substantially reduce the time and energy available for research. Administrative duties, teaching obligations, and activities with limited relevance to research frequently occupy time originally designated for academic work, trapping faculty members in a high-pressure, low-efficiency work cycle. One participant remarked: *“In addition to teaching, I have to handle all sorts of administrative forms, event planning, and evaluation preparations. Research requires deep focus, but my time is fragmented into small pieces, making it difficult to get into the right mindset. I feel increasingly distanced from my research.”* (Z20, Lecturer).

The combined pressure of teaching and research is further intensified when coupled with family responsibilities, exerting a sustained negative impact on academic productivity. Female faculty members are particularly vulnerable to this multi-directional strain, balancing caregiving, classroom teaching, and research duties. A female lecturer who had recently given birth described this conflict: *“My child is still very young and needs my care, but I have to teach three undergraduate courses in a single semester, in addition to managing the daily operations of the lab. My schedule is packed almost every day.”* (Z9, Lecturer). This cumulative pressure continuously erodes research time, making it difficult to conduct in-depth and systematic scholarly work.

In some universities, workload pressure also arises from numerous *ad hoc* assignments: *“Sometimes we are suddenly tasked with work unrelated to research, such as preparing for university anniversary events, hosting inspection delegations, or even setting up conference venues. As young faculty members, it is hard to refuse, but when these tasks pile up, research inevitably gets postponed.”* (Z17, Lecturer).

Participants widely advocated for the establishment of a more balanced allocation mechanism among teaching, administrative, and research responsibilities to reduce the drain of routine tasks on research potential and to prevent the persistence of a high-input, low-output cycle.

### Theme 5: authorship misattribution

Authorship misattribution is one of the most visible and personally impactful forms of CAB during the research output stage. Interviews revealed that such incidents often occur when a manuscript is nearing completion or ready for publication, involving intentional changes, downgrading, or complete removal of authorship positions in ways that distort the actual contribution ratio. Participants noted that these actions are typically covert and sudden, leaving the affected individual with no viable channel for appeal and compelling acceptance of the outcome.

In milder cases, authorship order changes occur just before submission: *“I was involved in every step, from topic selection and experiments to data analysis, and we had agreed from the start that I would be the first author. However, a few days before submission, I was suddenly told that the position would go to the project leader instead.”* (Z18, Lecturer). He emphasized that such a change disrupted existing plans and greatly diminished the value of years of research efforts.

In moderate cases, a researcher’s core contribution remains but is substantially reduced through adjustments in authorship order. One participant explained that she conducted most of the experiments and data processing, yet was listed only as the third author in the final publication, making it difficult to accurately reflect her actual contribution (Z7, Lecturer). Another participant with 8 years of research experience noted that these cases often stem from vague contribution metrics and informal negotiations. She had designed experiments and collected data for an interdisciplinary project, but at the submission stage, her authorship position was moved from second to fourth *“to maintain team balance,”* despite the project leader recognizing her substantial contribution (Z4, Lecturer).

In the most severe cases, authorship credit was entirely removed. One participant reported with strong criticism: *“I carried out the core experiments for the project and drafted the manuscript, yet when it was published, my name was completely gone.”* (Z15, Lecturer). She admitted that this experience had a long-term negative impact on her academic confidence and resulted in reduced participation in subsequent collaborations.

These accounts indicate that authorship misattribution represents a decisive blow to research output, directly undermining a scholar’s academic visibility and citation potential. It also creates cascading consequences within institutionalized evaluation processes such as research record compilation, academic rank promotion, and project competition. Such practices erode researchers’ motivation to remain committed to high-quality work and foster distrust within research teams.

### Theme 6: reputation manipulation

At the stage of research dissemination and academic evaluation, control over academic discourse, evaluation platforms, and channels of information dissemination may indirectly shape a scholar’s image and credibility within the academic community. Such practices do not necessarily involve explicit denial of scholarly competence; rather, they operate through selective disclosure of information, deliberate opinion shaping, or partial evaluation to construct external perceptions. These actions leave the targeted individual with little room for appeal or rebuttal, resulting in reputational damage.

Interview evidence illustrates these dynamics:

*“At an academic annual conference, when my research was introduced, they only mentioned ‘in collaboration with a certain professor’s team,’ omitting acknowledgment of my role as first author.”* (Z19, Lecturer).

*“Some review committee members implied that my work lacked innovation, yet they had not read the entire paper. Once such an assessment circulates, it inevitably shapes perceptions of my research.”* (Z6, Lecturer).

*“When competing for a project, certain colleagues privately questioned the feasibility of my topic, and these doubts rapidly circulated within the faculty.”* (Z13, Lecturer).

These cases demonstrate that reputation manipulation can, in the short term, diminish collaborative opportunities and reduce the visibility of outputs, and in the long term, lower citation rates and academic recognition of high-value work, continuously eroding research motivation and investment in innovation. When authorship misattribution and reputation manipulation occur together, the decline in both the quantity and quality of research outputs follows a cumulative and frequently irreversible trajectory, further entrenching inequality within the academic ecosystem.

## Discussion

This study, drawing on the experiences of YMTs, developed a chain-exclusion model comprising entry restrictions, process constraints, and endpoint undermining, revealing that CAB permeates the entire research cycle (see Figure 1). Most participants were lecturers in the early stages of their careers, and their primary research areas were in basic medical and pharmaceutical sciences. Interviews indicated that those with lower academic ranks were more vulnerable to power dynamics and resource control from senior colleagues. This was particularly evident in DFC medical universities, where the long-term dominance of core teams exacerbates the challenges encountered by peripheral YMTs in accessing information, gaining project entry, and utilizing research platforms. The chain-exclusion mechanism operates in a progressive and mutually reinforcing manner, where disadvantages at the entry restriction undermine resource allocation during the process constraint and are subsequently amplified in the endpoint undermining of evaluation and dissemination. Over time, this dynamic imposes long-term constraints on access to both information and resources.

### Covert academic bullying chain-exclusion model

#### Entry restriction: initiating mechanisms of information withholding and institutionalized marginalization

In the entry stage, closed-circle information circulation and institutionalized marginalization intertwine, forming the starting point of CAB. While these practices maintain an appearance of institutional transparency, they rely on informal networks to enforce rules, creating structural advantages under the guise of formal fairness. This finding aligns with the view that access to resources depends on information availability (Zhou, 2022) and underscores the distinct nature of medical research in China, where institutional design combined with a relationship-oriented culture can perpetuate inequality without overtly violating formal regulations (Manuel et al., 2024; Li et al., 2025).

The intersection of gender and career stage markedly exacerbates entry-stage disadvantages. Interview data indicate that female scholars during childcare periods often face dual constraints from family responsibilities and teaching loads, making it more difficult to enter high-value project networks (Horta and Tang, 2023). Early-career academics, particularly lower-ranked lecturers, often lack both institutional seniority and informal support, making them more susceptible to exclusion by higher-ranking colleagues in resource allocation, information access, and collaboration opportunities. This pattern is often described as newcomers being suppressed by veterans. The dual disadvantage of age and rank limits early-stage research engagement and develops into long-term barriers to career development, entrenching structural inequalities within the academic community.

In addition, prior research suggests that the generational gap between early-career academics and senior colleagues, particularly regarding psychological characteristics and competitive orientations, substantially influences academic interactions and research outputs (Horta and Li, 2024; Wierenga et al., 2025). Early-career academics at an early stage of their academic careers are often driven by an achievement-oriented sense of “voracity” and tend to validate themselves through rapid outputs. While this motivation can enhance productivity, it may also divert attention away from sustained scholarly accumulation and long-term research planning, thereby limiting their capacity to address complex research problems. In contrast, senior researchers, by virtue of accumulated experience, are better positioned to respond to academic challenges more strategically. The lack of experiential capital places early-career academics in disadvantaged positions within resource allocation and academic collaboration, often contributing to feelings of isolation. Consequently, generational disparities impose sustained psychological and structural pressures on early-career researchers and may ultimately constrain academic innovation and research diversity.

#### Process constraint: dual constraints of access deprivation and workload pressure

In the process stage, the combined effects of resource deprivation and workload pressure significantly limit sustained engagement in research. The heavy reliance of medical research on funding, specialized equipment, and clinical resources makes long-term monopolization by core teams a major barrier for peripheral YMTs (Vivek et al., 2025). Participants reported that peripheral faculty members often receive only temporary or low-priority resource access, undermining research stability and continuity. This finding aligns with studies on online health communities showing that physicians with low network centrality have limited influence (Wang H. et al., 2025).

For lower-ranked academics, these pressures are particularly acute, as they receive the lowest priority in resource allocation, lack stable access to experimental platforms and clinical resources, and are assigned large volumes of low-value administrative tasks. In addition, the substantial increase in teaching, administrative, and *ad hoc* non-research tasks fragments research time, resulting in piecemeal project progression. This phenomenon aligns with social network analysis (SNA) findings that peripheral members with low centrality face difficulties in bridging structural holes (Baek and Bae, 2019). Attempts to bridge these structural holes and act as “network brokers” can cause professional burnout and discourage engagement in high-risk research pathways (Lee et al., 2024). Without targeted institutional interventions, the imbalance in resource and task allocation is likely to persist, driving research teams toward conservatism and reducing overall innovation capacity.

##### Endpoint undermining: combined undermining effects of authorship misattribution and reputation manipulation

In the endpoint stage, the combined effects of authorship misattribution and reputation manipulation significantly diminish the visibility and academic recognition of research outputs. Changes in authorship order, dilution of contribution proportions, and removal of authorship rights create discrepancies between the academic record and actual contributions. Reputation manipulation shapes unfavorable perceptions through selective disclosure, partial evaluations, and informal opinion building (Khan and Tozal, 2025). These practices may appear in the early stages of an academic career and often become more prevalent over time (Savchenko and Rosenfeld, 2024). Young scholars have limited bargaining power, and once their reputation is damaged, opportunities for disseminating and citing their work are reduced, while subsequent project applications, academic rank evaluations, and cross-team collaborations are obstructed (Hall, 2023).

These findings confirm the relevance of academic reputation theory in explaining CAB’s impact (Savchenko and Rosenfeld, 2024) and suggest that the dual effects at the endpoint stage may generate long-term structural risks. These risks include topic convergence, suppression of innovative directions, and attrition of YMTs, ultimately reducing the diversity and public value of the academic community. Therefore, intervention strategies should prioritize governance of authorship practices and protection of academic reputation as essential measures to safeguard the visibility and career sustainability of young scholars.

### Logic of institutional centralization and relationship culture

The formation of CAB is closely linked to China’s highly centralized research management system and deeply embedded relationship culture. Research resource allocation depends on administrative rank and organizational affiliation, with funding, equipment, and platforms concentrated in public universities and core hospitals, distributed through institutionalized channels such as multi-tiered approvals, project selection, and expert recommendations (Li et al., 2022). In DFC medical universities, this centralization is more pronounced, with high-quality resources and policy benefits heavily skewed toward these institutions, granting structural advantages in research platforms, affiliated hospitals, and vertical funding (Jing and Hua, 2022).

However, concentrated resources intensify internal competition. Long-term monopolization of key platforms and high-value projects by core teams raises entry barriers for peripheral YMTs in information acquisition, project access, and platform use, with information withholding and opportunity exclusion occurring more frequently. By contrast, ordinary institutions have fewer total resources, but internal competition is more diffuse and thresholds for research collaboration and project access are lower (Liu et al., 2022). China’s interpersonal networks, mentorship cliques, and “informal apprenticeship” systems are particularly prominent in DFC medical universities. This mode of distributing information and opportunities relies on informal relationship networks, maintaining an appearance of openness while constructing invisible priority channels (Wang F. et al., 2025).

The intertwining of institutional and cultural factors further concentrates resources and opportunities at the center, making peripheral scholars more susceptible to personal relationships and informal evaluations in research output, authorship, and reputation. Research in China heavily depends on large teams, while smaller teams have experienced a marked decline in output (Liu et al., 2021). This “core–periphery” hierarchy reinforces the flow of resources and talent toward the center (Tian et al., 2024) and differs significantly from Western research systems, which rely on open applications, peer review, and diversified evaluation, providing fertile institutional ground for the persistence of CAB.

International literature has primarily focused on overt conflicts, gender discrimination, or direct interpersonal exclusion (Di Marco et al., 2021; Mukherjee, 2022), with discussions of early-career scholars centered on power asymmetry and unequal resource allocation during entry into academia (Habibie, 2022; Hearn and Husu, 2019). In engineering, life sciences, and social sciences in Europe, North America, and East Asia, early-career scholars frequently face the filtering effects of informal networks on information and opportunity distribution, path dependence on reputation and platform resources, and cumulative amplification of early disadvantages by evaluation systems (Fox and Nikivincze, 2021; Franzoni and Stephan, 2023). However, these studies often focus on single stages and lack systematic analysis of how institutional inertia interacts with implicit rules.

This study identifies two prominent differences in China. First, in DFC universities, YMTs face higher entry thresholds for academic participation, which, beyond equipment and funding requirements, include regulatory steps such as ethics reviews, clinical permissions, and patient case access. Second, academic exclusion significantly limits YMTs’ ability to improve network position and resource access through short-term strategies, whereas in non-DFC institutions, such obstacles are less severe. These differences indicate that YMTs in DFC universities face greater difficulty overcoming initial disadvantages early in their careers.

These structural characteristics contribute to the high stability and long-term persistence of chain exclusion in medical research. Accumulated regulatory steps, irreplaceable resources, fixed team roles, and closed-loop coupling between research output, reputation, and funding further concentrate power within networks. On this basis, the study reveals the full-chain mechanism across the entry, process, and endpoint stages and, through multidimensional analysis, elucidates its entrenching effects and far-reaching impact on the academic ecosystem.

### Intervention strategies and adaptive pathways

This study questions the assumption that scientific work functions within an ethically neutral or self-regulating environment. Despite the presence of formal regulatory mechanisms within academic institutions, practices such as covert exclusion, harassment, and violations of academic rights continue to persist, largely because covert academic bullying is embedded within informal relational networks and discretionary organizational processes that evade conventional oversight. Accordingly, rather than proposing a single regulatory instrument, this study puts forward a process-oriented governance perspective that targets key stages of the research cycle, including entry, process, and output, with the aim of reducing structural discretion, enhancing transparency, and strengthening procedural accountability. Within this framework, regulation is understood as a dynamic process of continuous alignment among institutional design, evaluative practices, and academic culture.

Intervention measures for different stages of the chain-exclusion mechanism should align with China’s centralized management system and deeply rooted relationship culture in medical research. In the entry stage, information asymmetry and institutionalized marginalization are the primary barriers faced by YMTs. To address these barriers, establishing a unified information platform, implementing open competition procedures, and optimizing anonymous review mechanisms are recommended. In DFC universities, priority channels for YMTs to access high-value projects and platforms should be introduced to mitigate initial competitive disadvantages.

In the process stage, resource deprivation and workload pressure are the main constraints on sustained research engagement. Drawing on the early career grant model (Mughal et al., 2021), YMTs could receive independent budgets, priority equipment access, and stable guarantees for experimental or clinical resources. Performance and workload management could also reduce non-research administrative tasks, preventing excessive fragmentation of research time.

In the endpoint stage, combined authorship misattribution and reputation manipulation undermine academic visibility and recognition. Optimizing evaluation and promotion systems, introducing contribution statements, and establishing third-party arbitration mechanisms could safeguard research attribution and support the sustained accumulation of academic influence.

As institutional reforms require time, adaptive individual-level strategies can also play a role. Such strategies include expanding cross-institutional and international collaboration networks, leveraging online academic platforms to build reputational capital, and integrating career planning, research management, and resilience development to enhance adaptability and stress tolerance. Through strategic topic selection and cultivation of supportive peer networks, YMTs can maintain research continuity and secure alternative resources to sustain scholarly development.

### Research significance, theoretical contributions, and practical implications

This study carries substantial theoretical and practical significance. Theoretically, it integrates perspectives from organizational psychology, labor sociology, and resource dependence theory to construct a chain-exclusion model comprising the stages of “entry restriction–process constraint–endpoint undermining.” This framework elucidates how covert power asymmetries and informal institutional mechanisms systematically shape academic behavior and productivity, thereby deepening the structural understanding of workplace bullying.

Empirically, focusing on young medical teachers in China’s Double First-Class universities and adopting phenomenological and social-constructivist approaches, this study reveals how resource control, hierarchical relationships, and evaluation systems collectively influence academic development and professional identity. These findings offer new empirical insights into the hidden conflicts that arise during the early stages of academic careers.

Practically, the findings indicate that universities and affiliated hospitals should acknowledge the potential impact of covert academic bullying on research integrity and clinical education. Institutions are encouraged to establish transparent and equitable governance and grievance mechanisms, refine authorship and workload policies, and enhance psychological safety and leadership ethics in faculty training, thereby fostering the academic development of young teachers and improving clinical education quality.

### Limitations and future directions

This study has several limitations. First, the sample was drawn primarily from 12 DFC medical universities, which may limit the generalizability of the findings. Future research should expand the scope to include other general institutions. Second, although this study focused on the perspectives of YMTs, incorporating the views of administrators, senior faculty, and other stakeholders in future research would yield a more comprehensive understanding. Third, although in-depth interviews yielded rich insights, the data may be affected by self-reporting bias, influenced by recall bias and social desirability effects. Subsequent research could adopt a mixed-methods approach integrating document analysis, social network analysis, and multi-group surveys for triangulation, thereby enhancing the robustness of the conclusions. Nevertheless, this study offers an important empirical foundation for understanding CAB mechanisms among YMTs and their impact on academic productivity.

## Conclusion

This study investigates the manifestations, mechanisms, and impacts of CAB on academic productivity among YMTs in China’s Double First-Class universities. Using phenomenological and social-constructivist approaches, the study develops a chain-exclusion model consisting of “entry restriction, process constraint, and endpoint undermining” to illustrate how covert power dynamics and informal institutional mechanisms shape academic behavior and development.

The findings indicate that young scholars face information barriers, resource constraints, and institutional marginalization in the early stages of their careers, experience excessive workloads and unfair evaluations during research, and encounter authorship manipulation and reputation suppression in the output stage. These covert mechanisms collectively limit academic agency, suppress innovation, and constrain professional growth.

Theoretically, this study broadens the structural perspective of workplace bullying research and enhances understanding of hidden conflict mechanisms within organizational culture and resource dependence. Practically, the study recommends that universities implement transparent and equitable governance systems, improve research resource allocation and authorship practices, and promote psychological safety and ethical leadership to cultivate a fair and supportive academic ecosystem.

## Data Availability

The datasets presented in this article are not readily available because the dataset contains sensitive interview information and cannot be made publicly available. However, de-identified data may be obtained from the corresponding author upon reasonable request. Requests to access the datasets should be directed to ZW, wuzaihao@xhu.edu.cn.
